# Chinese Regional Differences and Commonality in Field-Independence and Field-Dependence: An Implicit Biculturalism Model

**DOI:** 10.3389/fpsyg.2022.731722

**Published:** 2022-05-23

**Authors:** Wenli Liu, Zhaobin Dai, Shiwei Yang, Sik Hong Ng, Xiaocui Zhang, Shenli Peng

**Affiliations:** ^1^College of Education, Hunan Agricultural University, Changsha, China; ^2^Department of Psychology, Renmin University of China, Beijing, China; ^3^Medical Psychological Center, The Second Xiangya Hospital, Central South University, Changsha, China; ^4^Medical Psychological Institute of Central South University, Changsha, China; ^5^National Clinical Research Center for Mental Disorders, Changsha, China

**Keywords:** implicit biculturalism, culture priming, culture mixing, individualist/analytical cultural-cognitive system, collectivist/holistic cultural-cognitive system

## Abstract

Comparative studies of cultural-cognitive systems in China have stressed differences between northern and southern regions, with less attention paid to inter-regional commonality. This study proposes an implicit biculturalism model to rectify the diversity bias. The model posits that Chinese in both regions have internalized the same two cultural-cognitive systems but have organized them differently. For northerners, the individualist/analytical system (indicated by field-independence) is more dominant and chronically accessible than the collectivist/holistic system (indicated by field-dependence); for southerners the hierarchical order is reversed. The more dominant system would normally manifest in everyday life as the default situation, but the less dominant system could be activated through cultural priming. Both field-independent northerners (*N* = 46) and field-dependent southerners (*N* = 46) were assigned randomly into individualistic and collectivistic priming conditions and then tested with the Embedded Figure Test (EFT). The results indicated field-independent northern Chinese changed their EFT performance to be field-dependent under collectivism priming, and field-dependent southern Chinese changed their EFT performance in the field-independent direction, albeit to a less extent, under individualism priming. Generally, these results supported the implicit biculturalism model, which provides a more nuanced understanding of the question of “Who are the Chinese in Chinese psychology?”

*“… we invite Chinese psychology investigators to answer two hard questions: who are the Chinese in *your* Chinese psychology; what is Chinese about *your* Chinese psychology?”*

Ying-yi Hong, Yung Jui Yang, and Chi-yue Chiu (2010, pp. 21–22).

## Introduction

In cultural and cross-cultural psychology, Chinese participants are often grouped together as if they were a homogeneous and representative group for comparison with other cultural groups (Nisbett et al., 2001; Bond et al., 2004; World Values Survey, 2018). This research practice leaves open the question of subcultural diversities nested within Chinese culture (Huo and Randall, 1991). A similar question of subcultural diversity applies to cultural and cross-cultural studies more generally, and is not confined to the study of Chinese (Yamawaki, 2012; Oyserman, 2017). Examples of subcultural diversities are ethnic and – importantly as this is the focus of the present study – regional diversities among Han Chinese, the most populous ethnic group in Mainland China. (Unless stated otherwise, Han Chinese will be referred to by the shorthand form “Chinese”). Below we first review regional diversities that seem to be distributed along a north/south divide, such that northern Chinese are relatively stronger in individualism, independent self-construal, analytic cognition, and field independence, in contrast to southern Chinese’s stronger collectivism, interdependent self-construal, holistic cognition, and field dependence. Afterward, we propose an implicit biculturalism model to integrate both diversity and commonality between northern and southern regions.

### Psychological Diversities in Northern and Southern Regions of China

In their seminal research, Talhelm et al. (2014) attributed subcultural diversities in analytic/holistic cognitions and independent/interdependent construals of self to wheat- and rice-growing modes of subsistence. This wheat-rice theory led to empirical studies that attempted to locate the two modes of subsistence in various regions in China. A broad north-south divide emerged showing that Chinese in northern regions (along and north of Yellow River) were more analytic thinking and independent, whereas their counterparts in southern regions (along and south of Yangtze River) were more holistic in reasoning and interdependent in self-construal. Recently, a series of studies (Talhelm et al., 2018; Xu and Xin, 2019; Zhu et al., 2019; see Talhelm, 2020, for a review) have confirmed and manifested the north-south difference in China. Consistent with the north-south regional distribution of psychological differences are other research findings on individualism and collectivism (Ma et al., 2016) and field-dependence and -independence (Peng et al., 2018). Together, these studies point to a northern Chinese cultural-cognitive system featuring analytic and field-independent cognitions, independent self-construal, and individualism; and a southern Chinese cultural-cognitive system featuring holistic and field-dependent cognitions, interdependent self-construal, and collectivism.

The two north-south regional cultural-cognitive systems can be grounded in international cultural and cross-cultural psychological research. Most relevant to our present purpose is the early research on field-dependence and -independence. These psychological concepts refer to the difficulty or ease in separating an object from its surrounding environment (field) or in overcoming an embedding context (Witkin, 1967). Their measurement does not have to rely on questionnaire or self-report, but can be more objectively observed from participants’ performance on a cognitive/perceptual test such as the Embedded Figures Test, the Rod-and-Frame Test, and the Body Adjustment Test. Field-dependent individuals would take more time and make more mistakes than field-independent individuals would. The perceptual environment and mode of subsistence have considerable effects on the development of field-dependent and -independent cognitive styles (Berry, 1976; Uskul et al., 2008). For example, compared to sedentary farmers, hunters and gatherers would have to develop high field-independent abilities in order to find food and navigate in unfamiliar terrains. On the other hand, sedentary farmers would have to develop high field-dependent abilities in order to attend to their complex, interconnected social environment and maintain close work coordination with a large social network.

In more recent studies, Nisbett and his associates have proposed that field-independence can be considered as one part of a broader psychological construct called analytic thought, field dependence as a part of holistic thought, while still retaining their distinct conceptual, theoretical, and measurement features (Nisbett et al., 2001; Nisbett, 2003). These and other studies of culture and cognition point to two broad cultural-cognitive systems comprising, in one, analytic cognition (including field independence), individualism and independent self; and in the other, holistic cognition (including field dependence), collectivism and interdependent self (Kühnen et al., 2001; Nisbett et al., 2001; Heine and Norenzayan, 2006; Varnum et al., 2010; Miyamoto, 2013). Building on these studies, Oyserman (2017) summarized the two systems in terms of, first, an individualist mindset (individualism and independence) and associated analytic processing style, and second, a collectivist mindset (collectivism and interdependence) and associated holistic processing style. The author credited these two systems as two of the three “core themes” of cultural and cross-cultural psychology. In the present study, they will be referred to as individualist/analytical and collectivist/holistic systems, respectively. Against the backdrop of this body of international research, it would appear that the two cultural-cognitive systems are represented within the border of Mainland China, and distributed along a north-south axis. This is similar to the dual representation of analytic and holistic thoughts in Europe, distributed along a west-east axis in which Western Europe is more analytical and East/Central is more holistic (Varnum et al., 2008).

The Chinese regional studies reviewed above are useful in uncovering and describing broad regional differences. Our present study is not intended to uncover more regional differences, but to explore inter-regional commonalities that may lie underneath inter-regional diversity. For this purpose, we develop a model of “implicit biculturalism” based on a consideration of culture mixing that has been going on in the long history of China. Implicit biculturalism opens up the opportunity for applying culture priming to reveal inter-regional commonality.

### Implicit Biculturalism, Culture Mixing, and Culture Priming

Biculturalism refers broadly to the co-existence of two internalized cultures within the same individual (LaFromboise et al., 1993). It is a complex construct (Schwartz et al., 2017). Implicit biculturalism can be thought of as the shadow of explicit biculturalism. The latter is biculturalism that has been purposely sought out and marked as such by the researcher, for example, bicultural Chinese American (Benet-Martínez et al., 2002; Kim and Hou, 2016). When a sample of Chinese individuals is not explicitly marked as bicultural, it conveys the impression that the Chinese are monocultural, when in fact they may be implicitly bicultural embodying a second cultural-cognitive system that co-exists with but in the shadow of a relatively more dominant system. For example, although interdependent self-construal was the relatively dominant cultural-cognitive system of Hong Kong Chinese, independent self-construal was co-present, though weaker. The scores of interdependent/independent selves found in three separate studies were, respectively, 3.53/3.41 (Kwan et al., 1997), 5.67/5.00 (Singelis et al., 1999), and 5.62/5.29 (Ng and Zhu, 2001). The interdependent/independent differences are in fact relatively small in all three studies.

Culture mixing between northern and southern Chinese has been going on for millennia through trade and commerce, competition and conflict, and large-scale resettlement of people away from regions (mostly in north China) torn by internal war or political strife to regions of relative peace, mostly in south China (Ge, 1997). Apart from internally induced culture mixing, external culture mixing with foreign cultural groups has occurred along the old silk routes and through periodic invasions by powerful, highly individualist and independent prairie nomads north of the Great Wall, who have left behind a pool of diverse cultures, initially in north China and slowing finding their way south through internal culture mixing (Gao, 2010). A vivid illustration of culture mixing can be seen from the siting of dynastic capitals in and their frequent relocations to different regions. For example, the Song dynasty (960 –1,279) initially sited its capital in Bianjing (now Kaifeng) to consolidate its control of the traditional cradle of Chinese civilization along the Yellow River. Later it retreated south of Yangtze River under foreign invasions led by the Jin army from the north, taking with it a fleet of elite northern Chinese and a huge exodus of the population. Elite and popular cultures from north China migrated to south China, where the dynasty reincarnated itself as Southern Song (1,127–1,279) and relocated the capital to Lin’an (now Hangzhou). The resultant mixing of northern and southern cultures facilitated breakthroughs in agricultural, military and maritime technologies as well as commerce (Ge, 2005), boosted by a relatively rapid increase in population size and density in south China (Wu, 2010).

From this implicit bicultural perspective, northern Chinese should not be regarded as mono-culturally individualist/analytic, and those in the South are not mono-culturally collectivist/holistic. Instead they probably have internalized both cultural-cognitive systems, albeit in different hierarchical order. For northerners their individualist/analytic system is more dominant, more chronically accessible, than their collectivist/holistic system and hence it is the former system that would normally manifest in everyday life. Nonetheless, their second collectivist/holistic system has also been internalized, albeit in the shadow of the first, and would be “called out” by situational factors such as collectivism priming (see below). Conversely for southerners their collectivist/holistic system is the more dominant, chronically accessible one that would normally manifest in everyday life, but their second, less dominant individualist/analytic system would come out when individualism primed (see below).

Culture priming is an experimental procedure that uses situational cues to prime (to activate, stimulate, trigger, etc.) a particular cultural frame or knowledge structure that has been internalized or acquired by an individual (Hong et al., 2000). One commonly used priming procedure is to expose participants to pictures that are iconic of an individualist or collectivist country, another is to focus their thought on differences or similarities with family and friends: “think of how different you are from family and friends” (individualism) or to “think of how similar you are to family and friends” (collectivism). There are many other procedures (see Oyserman and Lee, 2007). Culture priming provides researchers with a useful experimental method that supplements traditional descriptive methods of cross-cultural comparison, and to move research from finding differences between cultures to exploring psychological mechanisms that may underpin the differences (Heine and Norenzayan, 2006). It has become widely used in cultural and cross-cultural psychology (Oyserman and Lee, 2008) as well as cultural neuroscience (Han, 2017).

Culture priming is expected to produce an assimilation effect by shifting behavior in the direction of the primed cultural frame/norm. However, this would only work if the cultural frame/norm has already been internalized and become accessible; if not, then there is nothing to be primed. The presence of assimilation would therefore allow researchers to infer the existence of a cultural frame/norm that may remain implicit under the shadow of a relatively dominant cultural frame (Ma et al., 2016). Studies by Kühnen and his associates illustrate this point. Participants in Kühnen, Hannover, and Schubert’s culture priming study were German university students. They were, according to another study, equally field-independent as their American counterparts and significantly more so than their Russian or Malaysian counterparts (Kühnen et al., 2001). As the authors did not explicitly designate them as bicultural, one might assume that they were mono-cultural in having only an independent construal of self (as indicated by their field-independence). However, this was not the case according to the results of four culture priming experiments (Kühnen et al., 2001). Compared to German students in the independent self-construal priming condition, those in the interdependent self-construal priming condition were more field-dependent. The fact that the interdependent prime was able to trigger an assimilation effect (increased the field-dependence score) indicated the presence of an internalized interdependent self-construal that co-existed with the independent self-construal.

### Hypotheses

The study reported below applied the research strategy of Kühnen and his associates to test implicit biculturalism of northern and southern Chinese. Participants were selected from the bigger sample in Peng et al.’s (2018) survey to represent northern and southern Chinese who were, respectively, field-independent and field-dependent. Three months after the survey, they were either individualism- or collectivism-primed. The main hypotheses based on the model of implicit biculturalism were (1) field-independent northern Chinese would become field-dependent under collectivism priming, whereas (2) field-dependent southern Chinese would become field-independent under individualism priming.

## Materials and Methods

### Participants and Experimental Design

Participants in the present experiment were 92 college students drawn from an earlier survey (Peng et al., 2018) using the extreme groups approach (Preacher et al., 2005). The survey covered 593 students in several colleges in Mainland China, 259 of whom were born in southern regions of China and the rest were born in northern regions. All had normal or corrected-to-normal vision, and reported no psychiatric or neurological diseases, and no alcohol or drug addiction. Birthplace in China was a reliable marker of regional influence as Chinese people generally remained in their birthplace to live and study for at least 18 years until they come of age for work or higher education, which could be indicted from the ID number of each participant. Participants of the previous (and the present) study were all college students, hence the years they stayed in the living places were not significant different from each other, as well as their age (*p*_*s*_ > 0.05).

A main effect of birthplace was found in the survey confirming that northerners were field-independent (higher EFT scores) and southerners were field-dependent (lower EFT scores). For the purpose of the present experiment, 46 of field-independent northerners (20 males) and another 46 field-dependent southerners (19 males) were selected from the survey, respectively (mean age = 21.25 years, SD = 1.87 years). They were randomly assigned to the individualism and collectivism priming conditions in equal numbers (the gender was balanced). Participants’ age did not differ between individualism (*M* = 21.7) and collectivism (*M* = 20.9) priming conditions, *t* (91) = -.81, *p* = 0.92. As the hypotheses were predicated on pre- and post-priming comparison within each priming condition rather than between priming conditions, a control group receiving neutral priming was deemed unnecessary for hypothesis testing. Even for comparison between priming conditions, researchers have seldom added a control group because of the ambiguity it would bring to data analysis (Oyserman and Lee, 2008), except for specific theoretical purposes such as the simultaneous testing of the assimilation and contrast effects of priming (Ng et al., 2016).

### Embedded Figures Test

The EFT was a timed paper-and-pencil performance test that had been revised and thoroughly pilot tested to suit Chinese participants (Meng and Chang, 1988). The task was to locate and trace the correct simple figure that was embedded in a complex figure. There were ten such tasks in each of two test sessions. Test reliability based on the correlation of correct answers between the two sessions was *r* = 0.90, *p* < 0.05. Validity was tested by correlating the EFT with the Rod-and-Frame test (*r* = 0.49, *p* < 0.05). Peng et al. (2018) has already applied the test to their survey and developed clear instructions on how to perform the task, which was in line with previous studies (e.g., Hao et al., 2013). In the present experiment, participants had to complete as many of the nine tasks in each session within 4 min, which was timed to suit university students. The total correct answers (original score) in the two sessions could vary from 0 to 20.

### Culture Priming

The culture primes in Hong et al’s. (2000) study were used for the present experiment and these administered via Microsoft PowerPoint. Participants in the individualism-primed condition viewed six icons of American culture, each presented on a different slide. Those in the collectivism-primed condition viewed six icons of Chinese culture. They were required to name the object presented in each picture and write a sentence to describe it. The appropriateness of this procedure for Mainland Chinese has been widely tested (Sui et al., 2007; Ng et al., 2010, 2016). We expected that individualism priming would facilitate a mode of thinking in which attention is directed to individual objects separated from each other and from their context. Consequently, it would induce a more field-independent cognitive mode, indicated by a high number of correct EFT answers. Conversely collectivism priming would facilitate a mode of thinking in which attention is directed to the relations of objects to their context, and consequently would induce a more field-dependent cognitive mode that would be indicated by a low EFT score.

### Experimental Procedure

The experiment was conducted 3 months after the survey. This period of time was deemed to be long enough to minimize the possibility that the EFT exercise in the survey might affect participants’ EFT performance in the experiment. Given the confirmed stability of cognitive style (Witkin et al., 1967), participants’ EFT scores in the survey would remain reliable after 3 months for comparison with their scores obtained in the present experiment. In the experiment, participants first completed the priming exercise and immediately afterward completed the EFT as described above. For the priming procedure, as mentioned above, both field-independent northerners and field-dependent southerners we assigned into the two priming conditions, resulting in 23 northerners and 23 southerners in individualistic priming condition and 23 northerners and 23 southerners in collectivistic priming condition.

### Data Analysis

To test the two hypotheses, planned contrasts were carried out to compare the pre- and post-priming scores of northerners in the collectivism-primed conditions (Hypothesis 1), and of southerners in the individualism-primed condition (Hypothesis 2). The MS_error_ term for constructing the two planned contrasts was derived from a three-way mixed ANOVA comprising Region (north vs. south China), Prime type (individualism- vs. collectivism-primed) and Test time (pre- vs. post-priming). Note that a significant three-way interaction effect would not be necessary for *planned* contrasts (Winer, 1991). Two sets of ANOVA and planned contrasts were conducted, based, respectively, on the original EFT scores and standard scores transformed from the original scores. The original EFT scores would test whether or not northerners would significantly lower their post-priming scores in the field-dependent direction (Hypothesis 1), and whether or not southerners would significantly improve their post-priming scores in the field-independent directions (Hypothesis 2). The transformed scores would allow a more stringent hypothesis testing by determining whether the primed changes might be interpreted as crossing from field-independence to field-dependence (Hypothesis 1), or from field-dependence to field-independence (Hypothesis 2). According to Meng and Chang (1988) the standard score (T) can be calculated from the following formulae:


$$T=t^{*}\,10+50,\text{and}$$

t = (O-N)/SD, in which O referred to the original score, N referred to the normative score, and *SD* referred to the standard deviation. As the numeric value of t was small, it was multiplied by 10 and increased by 50 to give the *T* value for presentation purposes. These authors have provided normative data for N and SD. They were, respectively, 9.86 and 4.45 for men, 9.69 and 4.89 for women, and 9.76 and 4.57 overall (see also Peng, 2014). The overall figures were used to calculate T scores in the present experiment. Compared to the original scores, T scores have the advantage that they can be used to set a normative threshold for field-independence. Given that the average EFT performance was around 55 (original score was 12) demonstrated by plenty of previous research (e.g., Jing, 2016; Peng et al., 2018), we set a threshold (*T* = 55) as the cut-off between field-independence and field-dependence, which has been adopted in Peng et al. (2018).

## Results

### Original Embedded Figure Test Scores (O_1_ and O_2_)

Table 1 (left portion) displays the means and SEs of the original EFT scores. A 2 (northerners vs. southerners) × 2 (individualism vs. collectivism priming) × 2 (O_1_ vs. O_2_) mixed ANOVA was conducted to extract the MS_error_ for constructing the contrasts, which was the usual practice with multiple comparison following significant overall test (Thompson, 1994; Hager, 2002), to test the hypotheses (MS_error_ = 1.14). Under collectivism priming, northerners responded by lowering their O score from 14.04 to 11.73, *F*(1,88) = 47.56, *p* < 0.001, Cohen’s d = −1.52. Under individualism priming, southerners responded by increasing their O score from 9.96 to 11.86, *F*(1,88) = 31.98, *p* < 0.001, Cohen’s *d* = 1.32. The highly significant planned contrasts supported both Hypotheses 1 and 2, details of which are shown in Table 2 (left portion).

**TABLE 1:** Means and standard errors of original and standard EFT scores stratified by region and prime type.

Region	Prime type	*N*	O_1_ mean (SE)	O_2_ mean (SE)	T_1_ mean (SE)	T_2_ mean (SE)
North	Individualism	23	14.04 (0.27)	14.09 (0.28)	59.25 (0.59)	59.29 (0.59)
	Collectivism	23	14.04 (0.27)	11.73 (0.29)	59.34 (0.56)	54.42 (0.77)
South	Individualism	23	9.96 (0.27)	11.86 (0.29)	50.45 (0.69)	54.24 (0.57)
	Collectivism	23	9.87 (0.27)	9.91 (0.29)	50.35 (0.45)	50.38 (0.44)

*O_1_, pre-priming original score; O_2_, post-priming original score; T_1_, pre-priming standard score; T_2_, post-priming standard score. Larger scores indicate higher field-independence.*

**TABLE 2:** Results of planned contrasts (original and standard EFT scores).

Source of variation	Difference (O_2_ – O_1_)	MS	*F*(1,88)	Difference (T_2_ – T_1_)	MS	*F*(1,88)
Pre- vs. post-priming scores of collectivism-primed northerners	−2.31	54.35	47.56***	−4.92	278.08	54.25***
Pre- vs. post-priming scores of individualism-primed southerners	1.96	36.54	31.98***	3.79	165.68	32.32***

O_1_, pre-priming original score; O_2_, post-priming original score, T_1_, pre-priming standard score; T_2_, post-priming standard score (see Table 1). Larger scores indicate higher field-independence. MS = mean squares. MS_error_ for testing (O_2_ – O_1_) = 1.14; MS_error_ for testing (T_2_ – T_1_) = 5.13, ***p < 0.001.

### Standard Embedded Figure Test Scores (T_1_ and T_2_)

**FIGURE 1:**
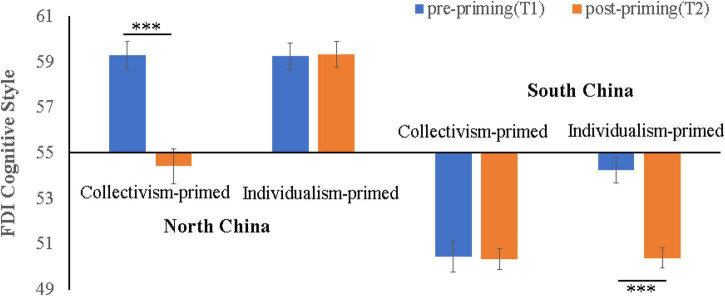
Effect of culture priming on standard EFT scores (T1 and T2). ***p < 0.001.

Table 1 (right portion) displays the means and SEs of the standard EFT scores. A 2 (northerners vs. southerners) × 2 (individualism vs. collectivism priming) × 2 (T_1_ vs. T_2_) mixed ANOVA was conducted to extract the MS_error_ for constructing the contrasts to test the two hypotheses (MS_error_ = 5.13). Note that *T* > 55 indicated field-independent, whereas *T* < 55 indicated field-dependent individuals. As shown in Figure 1, field-independent northerners responded to collectivism priming by lowering their T score from 59.34 (field-independent) to 54.42 (field-dependent), *F*(1,88) = 54.25, *p* < 0.001, Cohen’s *d* = −1.79. Hypothesis 1 was fully supported. Under individualism priming, southerners responded by increasing their T score from 50.45 (field-dependent) to 54.24 (still field-dependent), *F*(1,88) = 32.32, *p* < 0.001, Cohen’s *d* = 1.25. Although the change was significant and in the direction as predicted by Hypothesis 2, it failed to exceed the field-independent threshold of 55. Hence, unlike in the case of original scores, the standard scores provided only partial support for Hypothesis 2. Details of the planned contrasts are shown in Table 2 (right portion).

### Other Results

Table 1 shows other results of interest. Individualism priming did not change northerners scores at all – their O_2_ and T_2_ scores remained the same as their respective O_1_ and T_1_ scores. Neither did collectivism priming lead to any noticeable changes among southerners. These results raise intriguing questions.

## Discussion

Compared to the increasing attention on culture diversity, relatively less attention has been paid on the commonality across regions in China. The present study proposed an implicit biculturalism model to rectify the diversity bias but without over-emphasizing commonality. Instead of assuming that northern Chinese were individualist/analytical and southern Chinese were collectivist/holistic, the model posited that Chinese in both regions have internalized the same two cultural-cognitive systems as a result of culture mixing but have hierarchically organized them in different ways. For northerners, the individualist/analytical system is more dominant than the collectivist/holistic system; for southerners the hierarchical order is reversed. The more dominant system would normally manifest in everyday life as the default situation. It was this surface appearance showing regional diversity that was emphasized in reports by Talhelm et al. (2014) and others, to the neglect of the less dominant system. The latter, however, would manifest when primed (made psychologically salient) by situational cues that were culturally congruent with it. This core tenet of the model led to Hypotheses 1 and 2, which were tested by experimental priming results based on original and standard EFT scores. The standard scores complemented the original scores by providing a prior threshold for interpreting whether or not a particular EFT score was field-independent or -dependent.

Overall the results showed that apparently individualist/analytical northerners were indeed field-independent prior to culture priming. However, under collectivism priming, their EFT performance decreased significantly in original scores, indicating obvious reduce in field-independent tendency, and changed to the extent that could be interpreted as field-dependent when expressed in standard scores. As field-dependence was a part of the collectivist/holistic cultural-cognitive system, the results revealed the presence of this system in their mindset (Hypothesis 1 was supported). Second, southerners whose pre-priming EFT performance was field-dependent (indicative of their collectivist/holistic system), changed their EFT performance in the field-independent direction to reveal their second individualist/analytical system when individualism primed. Their post-priming EFT original score was significantly higher than their pre-priming original score in the predicted direction, but just fell short of the threshold required for field-independence when used standard scores. Hence this part of the results supported Hypothesis 2.

Below we discuss these and other results, reflect on their relevance to past and future research, as well as point out their limitations.

The two significant findings summarized above are consistent with the assimilation effect of culture priming that has been widely reported in the priming and biculturalism literatures (Hong et al., 2000; Cheng et al., 2006; Oyserman and Lee, 2007; Chen et al., 2008; Ng et al., 2016). Assimilation is said to occur when an individual shifts toward the cultural-cognitive system that has been primed, the precondition of which is that the relevant system must be present. The precondition in the case of northerners is their collectivist/holistic system, and in the case of southerners is their individualist/analytic system. These are, according to the implicit biculturalism model, the less dominant of the two systems of northern and southern Chinese, respectively. Since the predicted outcomes are significant, it would be reasonable to infer that the two systems are, as proposed in the implicit bicultural model, present in northern and southern Chinese.

In contrast to the assimilation effects on the less dominant system, there was no assimilation effect on the more dominant system. When individualism primed, northerners did not become more field-independent in the direction of their dominant individualist/analytical system, which would otherwise indicate assimilation; and southerners when collectivism primed did not become more field-dependent in the direction of their collectivist/holistic system, which would otherwise indicate assimilation. The absence of assimilation effect is not unusual (Heine et al., 2001; Oyserman and Lee, 2007). It is nonetheless intriguing in light of the presence of assimilation effects on the less dominant cultural-cognitive system. One possible explanation may be derived from the implicit biculturalism model and the ceiling/floor effect. According to the model the more dominant of the two systems is the default system normally “on show” in everyday life requiring no additional booster or intervention from experimental priming. This would be the case because the dominant system is a relatively well-scripted “cultural practice” shored up in the brain ready for cognitive service (Kitayama and Uskul, 2011). For northerners, ceiling effect may then kick in because their mean original EFT score prior to priming (over 14, see Table 1) was already close to the maximum possible end point of 20 in the EFT exercise. For southerners, their mean EFT original score prior to priming was around 9. Although this figure appeared to be well above the minimum possible end point of zero in the EFT exercise, the actual “floor” that would allow room for downward shift was not zero but well above zero because the participants were highly educated university students who were most unlikely to score zero in the EFT exercise. This explanation is in line with the argument that the priming information that is redundant with what people are chronically exposed to should have little impact (Heine et al., 2001; Peng et al., 2020).

The implicit biculturalism model affords a more comprehensive view of the north-south Chinese regional differences reported by Talhelm et al. (2014) and others reviewed earlier in this paper. Such differences were similar to the present north-south differences obtained under default situation prior to experimental intervention (priming). They were comparisons made only of the dominant cultural-cognitive systems of northern and southern Chinese. Hence, the present study goes beyond previous studies by confirming a more dynamic picture of the Chinese regional difference, consisted of a second, less dominant system untapped by the methodologies that those authors had used.

More generally the implicit biculturalism model offers an approach to the sobering questions posed by Hong et al. (2010, pp. 21–22) and quoted at the beginning of the present paper: “who are the Chinese in *your* Chinese psychology; what is Chinese about *your* Chinese psychology?” The model makes explicit the co-possession of two mindsets (collectivist/holistic and individualist/analytical) in the bicultural mindset of Chinese, regardless of whether they are northerners or southerners. The model by itself does not provided answers to Hong et al.’s (2010) questions, however, it shines a brighter light than monocultural mindset approaches does.

The model’s relevance to the understanding of Chinese is likely to increase in the future as the country is exposed to new waves of culture mixing. Internal migration in China has increased rapidly since 1990 (Lu and Xia, 2016), driving internal culture mixing in the process. External culture mixing through globalization and gǎigé kāifàng (“economic liberalization and opening to the world”) (Chen et al., 2008; Ng et al., 2011) will be further enriched by the “One Belt, one Road Initiative,” which will widen the scope of culture mixing to cover the cultures of Muslims and Africans, among others (Kohli, 2018). Younger generations in the future will have the opportunity of *early immersive* culture mixing (Martin and Shao, 2016) with these cultures from childhood onward, the implications of which for bicultural development and innovative orientation await research (Hao et al., 2016).

Several limitations of the present study should be noted. First, the sample of university students clearly limits the extent to which the findings can be generalized to the population. The ecological validity is further limited because the sample covers only a few of the typically northern and southern regions, each in small numbers. A more representative sample of adults are encouraged in future studies to acquire a reliable conclusion. Second, the EFT was used as the single measure of broad individualist/analytical cognitive-cultural system, which is far from desirable notwithstanding its ability in providing an objective measure of field-independence as a part of the system. Supplementary measures would be useful in future research, for example, the Framed Line Task (Kitayama et al., 2003) as an alternative, but similarly task-based, measure of field-dependence/independence, the Analysis-Holism Scale that surveys analytic versus holistic thinking tendency (Choi et al., 2007), and fMRI techniques that scan the bicultural brain (Ng et al., 2010; Oyserman et al., 2014). This and other measures that are more sensitive than the EFT in picking up priming effects would be particularly advantageous in re-testing Hypothesis 2, which has received only partial support from the standard EFT scores. This elicits an additional issue that the set of the threshold in our study is not frequently used in EFT research, which suggested the outcomes derived from the original scores was more reliable in our study. However, we remind the readers that the current results that when northerners and southerners veered away from their default tendency, their performance became indistinguishable from this average T (Table 1), supported our classification criterion. Fourth, the lack of participants’ living area information (urban/rural area) made it unlikely to investigate the possible urban-rural differences in individualism/collectivism (Heu et al., 2019) that might interfere the current findings. However, there is research that Chinese urban youth scored equally with rural youth on Framed Line Task (Huang et al., 2014), indicating there is no urban-rural difference in field-dependence/-independence. Given the inconsistent findings, more empirical evidence is needed.

## Conclusion

The present results showed that field-independent northern Han Chinese changed their EFT performance to field-dependent when exposed to collectivism priming, whereas field-dependent southern Han Chinese changed their EFT performance in the field-dependent direction under individualism priming. Overall, the results supported the implicit biculturalism model, according to which northern and southern Han Chinese have internalized the same individualist/analytical and collectivist/holistic cultural-cognitive systems as a result of culture mixing, but have organized them in different hierarchical order. Past research investigating differences in northern and southern Han China has stressed their respective dominant, default cultural-cognitive system to the neglect of their less dominant, submerged cultural-cognitive system. The current findings advance the understanding of the north-south Han Chinese regional differences and commonality, and will become even more relevant as the country is exposed to new waves of culture mixing (Peng et al., 2017).

## Data Availability Statement

The datasets presented in this study can be found in online repositories. The names of the repository/repositories and accession number(s) can be found below: http://osf.io/yj6u9/.

## Ethics Statement

The studies involving human participants were reviewed and approved by the Institutional Review Board of Hunan Agricultural University. The patients/participants provided their written informed consent to participate in this study.

## Author Contributions

SP and WL designed this study. WL, ZD, SY, SN, and SP collected and analyzed data. SP, WL, and XZ wrote the draft. WL, SN, SP, and XZ revised the manuscript. All authors contributed to the article and approved the submitted version.

## Conflict of Interest

The authors declare that the research was conducted in the absence of any commercial or financial relationships that could be construed as a potential conflict of interest.

## Publisher’s Note

All claims expressed in this article are solely those of the authors and do not necessarily represent those of their affiliated organizations, or those of the publisher, the editors and the reviewers. Any product that may be evaluated in this article, or claim that may be made by its manufacturer, is not guaranteed or endorsed by the publisher.
